# What makes stroke rehabilitation patients complex? Clinician perspectives and the role of discharge pressure

**DOI:** 10.15256/joc.2016.6.63

**Published:** 2016-03-04

**Authors:** Michelle L.A. Nelson, Elizabeth Hanna, Stephen Hall, Michael Calvert

**Affiliations:** ^1^Bridgepoint Collaboratory for Research and Innovation, Lunenfeld-Tanenbaum Research Institute, Sinai Health System, Toronto, ON, Canada; ^2^Daphne Cockwell School of Nursing, Ryerson University, Toronto, ON, Canada; ^3^Dalla Lana School of Public Health, University of Toronto, Toronto, ON, Canada; ^4^Bridgepoint Hospital, Sinai Health System, Toronto, ON, Canada; ^5^Faculty of Medicine, University of Toronto, Toronto, ON, Canada

**Keywords:** biopsychosocial factors, stroke, discharge pressure, inpatient rehabilitation, patient complexity

## Abstract

**Background:**

Approximately 80% of people who survive a stroke have on average five other conditions and a wide range of psychosocial issues. Attention to biopsychosocial issues has led to the identification of ‘complex patients’. No single definition of ‘patient complexity’ exists; therefore, applied health researchers seek to understand ‘patient complexity’ as it relates to a specific clinical context.

**Objective:**

To understand how ‘patient complexity’ is conceptualized by clinicians, and to position the findings within the existing literature on patient complexity.

**Methods:**

A qualitative descriptive approach was utilized. Twenty-three stroke rehabilitation clinicians participated in four focus groups.

**Results:**

Five elements of patient complexity were identified: medical/functional issues, social determinant factors, social/family support, personal characteristics, and health system factors. Using biopsychosocial factors to identify complexity results in all patients being complex; operationalization of the definition led to the identification of systemic elements. A disconnect between acute, inpatient rehabilitation and community services was identified as a trigger for increased complexity.

**Conclusions:**

Patient complexity is not a dichotomous state. If applying existing complexity definitions, all patients are complex. This study extends the understanding by suggesting a structural element of complexity from manageable to less manageable complexity based on ability to discharge.

## Introduction

Stroke patients are admitted to rehabilitation programs with multiple medical/functional and psychosocial issues [1–3]. Stroke occurs in isolation (no other chronic conditions) in only 6% of stroke survivors [4, 5], and patients have on average five other conditions [1, 3, 4]. Common psychosocial issues include marital and family stress, inability to return home, inability to return to work, decreased or limited social interaction, depression, and issues in adjusting to having a disability [6, 7]. Retrospective studies of stroke rehabilitation have found that multimorbidity increased rates of complications, led to longer hospital stays, and was negatively correlated with functional outcomes for the patient – increasing the cost and decreasing the efficiency of rehabilitation [3, 8, 9].

Increasing attention to the range of biopsychosocial issues people experience has led to the identification of ‘complex patients’ [10]. Complex patients could be described as those who, in addition to having multiple comorbid conditions, may experience other issues that impact their ability to self-manage or to benefit from healthcare interventions. To date, however, there is no single widely accepted and utilized definition of ‘patient complexity’. A wide range of terms such as ‘comorbidity’, ‘multimorbidity’, ‘co-occurring conditions’, and ‘complex chronic disease’ are used to describe this patient population – often synonymously [11]. Managing complex patients requires greater clinician effort, increased healthcare resources, and substantial family and community supports, but if healthcare systems and services are to be redesigned to better meet the needs of these patients, a better understanding of the complex patient population is needed [12].

An emerging body of work has focused on identifying and understanding complex patients through processes such as counts of comorbid conditions, weighted values based on clinical needs, assessing healthcare utilization, and incorporating psychosocial elements [13–17], with some researchers suggesting conceptual models and frameworks [10, 17]. These studies have identified key elements of patient complexity related to the meshing of two or more factors (disease states, socioeconomic status, coordination of care, family and environmental situations). Without a single conceptual framework or a unifying definition, and with some definitions of complexity excluding nonmedical dimensions, applying the term ‘patient complexity’ can be challenging [18]. In the absence of a unifying definition or single conceptual framework, however, applied health researchers have sought to understand the concept of ‘patient complexity’ as it relates to specific clinical contexts or situations [12]. Although attempts have been made to understand complexity from patient perspectives and experiences, [19], care needs [12], and from a policy and economic viewpoint [20], no work to date has explored the perspectives of rehabilitation clinicians who work directly with complex stroke patients. 

## Objectives

The purpose of this study was to: i) understand how ‘patient complexity’ is defined and conceptualized by stroke rehabilitation clinicians, and ii) position the findings within the existing conceptual literature on patient complexity.

## Methods

This study was conducted using a qualitative descriptive approach. After data collection and analysis, the researchers positioned the emerging findings within the existing literature on patient complexity. The study protocol received approval from the Joint Bridgepoint Health – West Park Healthcare Centre – Toronto Central Community Care Access Centre Research Ethics Board prior to any recruitment and data collection.

## Study setting

The study took place at two inpatient neurorehabilitation clinical units at a mid-sized complex rehabilitation hospital (with approximately 400 beds) in a large Canadian city. Patients were admitted from acute care hospitals within 10 days post stroke, for approximately 3–12 weeks of therapy. One clinical unit provided high-intensity, short-duration rehabilitation, and the other was a lower intensity, longer duration program. Patients at each unit received a variety of therapy services, dependent on patient needs and tolerance for rehabilitation activities. As a publicly funded hospital, costs of the programs were covered by the provincial healthcare budget. 

Professions on the clinical teams included nurses (both registered and registered practical nurses), occupational, physical and recreation therapists, physicians, social workers, pharmacists, speech language pathologists, dieticians, and spiritual care providers, as well as rehabilitation therapy assistants. Staffing varied according to shift, but daytime full-time equivalencies (FTEs) for health discipline and nursing staff were approximately 25 for each program. Nursing care was provided in shift work by a roster of providers; rehabilitation therapists were dedicated FTEs and specific staff members for each unit. Physician services were provided by both hospitalists and consulting physiatrists, none of which were dedicated full-time physicians for the neurorehabilitation inpatient units. 

## Participants

All members of the interprofessional teams practicing within two stroke programs at a complex rehabilitation hospital were invited to participate through existing e-mail distribution lists. Twenty-three stroke rehabilitation clinicians (12 nurses and 11 therapists: occupational therapists, physical therapists, speech language pathologists, recreation therapists, and rehabilitation therapy assistants) participated in one of four focus groups ­(FG1–4). This reflects approximately half of the therapists dedicated to the neurorehabilitation units, and about half of the nursing FTEs for the clinical programs. Although physicians (*n*=4) and administrative team members (*n*=6) were included on the e-mail invitation, none volunteered to participate. Due to recruitment procedures, rationale for nonparticipation was not provided; however, shift scheduling, patient care requirements, and other research opportunities likely impacted recruitment efforts and participation rates.

## Data collection

Four focus groups were conducted utilizing an interview guide developed from existing literature regarding complexity and/or stroke rehabilitation, and expert opinions. Additional questions were developed as data were collected and potential themes identified. Participants were encouraged to provide information beyond the scope of the interview guide as they saw relevant. 

All focus groups were facilitated by the same researcher with extensive experience in qualitative data collection approaches and methods. Each focus group was scheduled at the end or beginning of a regular shift schedule to maximize potential participation. Each focus group began with a global question: “Thinking of a stroke rehabilitation patient you would consider ‘complex’, what was it about that particular patient that made them complex?” Participant responses were posted on a portable whiteboard. Clarifying and exploratory questions regarding the characteristics were posed, and then participants were asked to delineate which of the characteristics were specific to stroke rehabilitation patients. Participants in FG2–4 developed their lists of characteristics individually, and then added their contributions to the whiteboard, which contained responses from all of the previous focus groups (aggregated). For the purpose of this study “theoretical saturation” was considered to be met when new information added only minor variations to the characteristics and subsequent definition of complexity.

## Data analysis

Data were analyzed in an iterative and reflexive format, using qualitative content analysis techniques, resulting in the simultaneous data collection/analysis process consistent with interpretive research approaches. Review of the collected data provided an overall sense of the meaning and a scope of the information collected, including an initial impression of the overall depth, credibility, and potential usefulness of the information. This assisted in determining whether theoretical saturation had been met or if additional focus groups were needed. 

Audio recordings of each focus group were transcribed and initially coded thematically, using word-processing software, based on the characteristics and categories created during the focus groups (posted by participants on the portable whiteboard). This allowed the research team to determine whether the participant-identified categories could be used as a thematic framework or what modifications were required. Consistent with qualitative content analysis, the research team subsequently expanded upon and created new categories to ensure all the characteristics identified were represented. Each transcript was then reanalyzed by two research team members using the revised framework. Data were analyzed for difference based on clinical program (low- or high-intensity rehabilitation units); however, participants held very similar perspectives regardless of clinical program. 

Study results were summarized in a descriptive summary of the content, organized to best contain and present the data collected. A final analytical activity mapped participant descriptions to existing conceptual frameworks and literature regarding patient complexity, looking for similarities, differences, and any extension of the work. 

## Results

Five elements of patient complexity were identified: i) medical/functional issues, ii) social determinant factors, iii) social/family support, iv) personal characteristics, and v) health system factors (Table 1). Due to the high degree of patient variability, it was difficult for participants to prioritize a specific factor as “*it is hard to say which of these factors are more (or less) significant, because something that is small for one patient may be huge for another patient” (FG2).*

## Medical and functional issues

Patients with several co-occurring medical and/or functional issues may not *“need acute medical care but they are still not ready to go home or to the community due to their physical condition. These patients with multiple diagnoses need active treatment from specialized disciplines”* *(FG2)*.

Patients with several pre-existing chronic conditions are complex, as noted: *“A patient is on dialysis and had visual issues, and now put a stroke on top of that… she used to do things by feel but now her hand is not working as well… that is complex” (FG2).* The impairments resulting from the stroke (e.g. impaired cognition, aphasia, and dysphagia) could also contribute to complexity by hindering participation in rehabilitation activities as noted by one participant: *“When you are working with people’s cognition, if you can’t communicate with them, you can’t do therapy. Put in aphasia then cognitive testing, language testing, their ability to follow directions in therapy – it is all affected, making them complex” (FG2).*

The sudden onset of stroke-related impairments was identified as one type of complexity for patients: “*If you’ve had a stroke, you are fine one day and then you’re not fine the next. These patients have cognitive changes, physical changes, and visual perceptual changes. That is a complex patient” (FG2).* Participants clearly stated, however, that at the rehabilitation program level it did not matter if the medical/functional conditions were pre-existing or resulting from the stroke, as all of the issues must be addressed by the clinical team in the rehabilitation process: “*When people come in, what makes them complex is this plus, plus, plus. So they have pre-existing conditions, now they [have] stroke-specific issues and these are compounded by new problems, all of which we have to address*” *(FG3)*. 

## Social determinant factors

The definition of complexity provided by the clinicians included more than the medical/function impairments, and took the whole life of the patient into account: *“It’s rarely just a stroke. We have to deal with everything they were dealing with before. Some people were dealing with job situations – you still have to deal with that. Now it has a stroke spin to it. We are often treating more than stroke*” *(FG1)*.

The discharge destination (i.e. home, long-term care, or outpatient rehabilitation) as well as the previous housing situation can contribute to complexity. Homelessness poses a particular challenge. Patient literacy, educational level, employment type and status with the corresponding financial aspects may contribute to complexity and must be addressed by the rehabilitation team.

The clinical teams must take into account whether the patient is likely to have access to both personal and public transportation, which was particularly problematic for patients who had cognitive rather than physical impairments: “*If someone is up and ambulatory and their vision is fine, but cognitively they are off – they are at a loss for a lot of things – like transportation. They can’t get ‘wheel trans’ [supportive transportation] because they are physically ok, but they are still very complex because they’ve got cognitive issues” (FG4).*

## Social/Family support

Social support was identified as a key theme related to complexity and successful discharge: *“Social supports are one of the number one things… if someone has a good social network, then we are able to do our work and help someone get home*” *(FG1)*. Family engagement can also either alleviate patient complexity, making patient care more manageable and less complex, or it can hinder treatment goals, compounding complexity: 

*Respondent: Sometimes the family does not have insight either.*

*Interviewer*: *Are you saying that family contributes to making people more complex?*

*Respondent:* *For sure…Or less complex. They may help us as well. They know more, and they provide a lot of emotional support and encouragement. If we don’t have that then we’re dealing with that patient on their own. And if there are other things like language…all those other things and no family support, then we’re really lost. And financially they can help, or they can troubleshoot and help us more” (FG2).*

## Personal characteristics/coping style

Personal characteristics can either exacerbate or mitigate patient complexity. Personal characteristics included age, degree of confidence, self-efficacy beliefs, motivation for improvement, denial, coping skills, and having a positive or negative attitude. *“One thing that can make things more or less complex is pre-morbid outlook on life or attitude about dealing with difficult situations and illness. I think people who see the glass half full can reduce complexity. Some people have a lot of deficits but they have a really good outlook, so it’s not going to affect them to the degree that it affects a person who doesn’t have such a positive outlook”* *(FG1)*.

## Health system factors

Patient complexity was also attributed to health system factors or the mismatch between the individual patient and the expected rehabilitation process or ‘flow’. Complexity is the interaction between medical/functional and social determinant factors, social support and patient characteristics that hinders discharge. Participants across the focus groups agreed: *“How all of these combine is the hard part – what they come in with, what they treat and issues around discharge, that is the complex part. All of these issues tie into the discharge process and for me the complexity is connected to the push to get patients discharged faster”(FG2). “So we have to identify the things that may be triggers for complexity because you are thinking about discharge right away*” *(FG1)*. *“The complexity (that has been increasing more recently) that I feel is connected to the push to get patients discharged faster. So before, when the length of stay was months, we had time to deal with all of the issues. Now that there is [a] push to get them out faster, to go home or to wherever they are going – so it’s the push [that] increases the speed at which we need to work which makes things so much more complicated, and especially if their recovery has not been moving as fast as we need to move” (FG3)*.

The disconnect between the acute healthcare system, inpatient rehabilitation, and community-based services was identified as a trigger for increased complexity. Shifting the length-of-stay target in one sector of the stroke system significantly influences the partners downstream: *“A systems thing that contributes to the complexity… we do have certain guidelines that are in place. And in terms of lengths of stay – there is pressure to get people moved along quicker…*” *(FG2).*

When asked what proportion of their patients would be considered ‘complex’ based on the identified factors (Table 1), participants responded that *“everyone is”* and that *“complexity is now the norm.*” When asked what differentiated the patients they initially identified as complex from other stroke rehabilitation patients, participants responded that “*thinking of the patients we’ve had and what hung them up at discharge is always this stuff [referring to the medical/social/system characteristics]. It’s always a combination. Very rarely do we get an easy discharge and that makes people more complex” (FG3).* Complex patients who are discharged in concordance with length-of-stay targets were therefore not considered to be as complex as those who could not be discharged in the timeframe dictated by evidence and best practice guidelines. 

## Definition not specific to stroke

After discussing each of the complexity characteristics (Table 1), participants determined that the multitude of health conditions and social factors present in complex patients are not specific to stroke*.* Of all the characteristics of complexity, only the ‘diagnosis’ of stroke was ultimately determined to be specific to stroke. Specific to stroke patients, however, was the immediate onset of several complexity factors from a single medical event: *“It’s so hard to separate stroke from any other neurological issue. None of the issues [pointing to the stickies on the board] are stroke specific, besides diagnosis. All of them apply everywhere and can apply anywhere” (FG4)*.

## Discussion

This exploratory study provides a rich description of clinician perspectives on what constitutes patient complexity in stroke rehabilitation, which had not been represented in the literature to date. Study results support current definitions and conceptualizations of complexity, but also provide insight into the operationalization of complexity definitions in stroke rehabilitation, highlighting a key systemic element of patient complexity. 

Not surprisingly, the patient characteristics identified in this study mirror the existing body of literature regarding comorbidity, multimorbidity, and the social issues experienced by stroke rehabilitation patients [3, 5]. In addition, many of the characteristics of complexity identified by participants also align with the characteristics already identified by authors of previous research and conceptual frameworks regarding complexity [17–20]. Existing conceptual frameworks such as the one generated by Schaink *et al.* [17] used scoping review results to identify five complexity components (medical, social, demographic, mental health, care system). Each of these components were noted within the presented study to be characteristics of the ‘typical’ complex stroke patient; resulting in the clinicians’ conclusion that complexity was the norm rather than the exception in stroke rehabilitation. Complexity characteristics such as language, access to community services, and mobility status are not unique to stroke patients; these characteristics are found in many other patient populations requiring rehabilitation. A key contribution of this work is, therefore, not simply an understanding of complexity from the perspective of stroke rehabilitation clinicians, but rather support for the development and application of a common, unifying definition rather than multiple definitions and conceptual frameworks based on patient population and/or clinical context. 

In the operationalization of the definition of complexity to the clinical context, a rehabilitation-specific element was identified. As noted, if simply applying existing indicators of complexity, every stroke rehabilitation patient would be considered complex. Clinicians identified a subset of patients who they felt were more complex, also seen as unmanageably complex, described as those who were more difficult to discharge in comparison to the ‘typical’ patient. Most notable were clinician concerns focused on length-of-stay targets, a systemic factor that is a particular focus in stroke rehabilitation as programs and services are being continually aligned with the Canadian Best Practice Recommendations for Stroke Care [21]. This systemic definition of complexity is built upon clinician perception of a mismatch between patient complexity characteristics and expected length of stay. This study result is particularly important given the increasing focus on shortening the length of hospital stays, leading to a potential system-fostered patient complexity. 

This research also suggests that some of the complexity factors (social/family support) may exacerbate or mitigate complexity, in turn either facilitating or hindering patient discharge. This suggests that the characteristics of patient complexity are not a checklist of items resulting in a dichotomous variable – complex or not – but rather may serve as a spectrum of characteristics that may make a patient more or less complex. Further understanding of this ‘complexity spectrum’ and its influence on care processes could facilitate alternative care pathways and supports for patients identified as ‘unmanageably complex’. 

This study was designed to serve as a foundational component for a larger program of research focused on complexity and stroke rehabilitation. The aim of the study was to understand complexity in the context of stroke rehabilitation, and support the development of future research studies. As a question-generating study, it was successful, and many questions have arisen regarding the use of study results to support practice changes. Correspondingly, a limitation of the study is the inability at this stage to recommend practice changes. The study has provided an initial understanding of what may be occurring related to complexity and stroke rehabilitation, but does not provide grounds for recommendations for practice settings. Additional research, undertaken in partnership with clinical settings, is needed to determine what effect these results may have in improving rehabilitation services. Key questions remain: Is the perceived mismatch between patient characteristics and system requirements real? If so, how are stroke rehabilitation teams managing complexity within these time constraints? What practice-level strategies are being employed to reduce complexity and facilitate timely discharge from the rehabilitation units? How might these be scaled to other programs and jurisdictions?

## Conclusions

Complexity is not a dichotomous state – complex or not. Simply applying existing complexity definitions, all stroke rehabilitation patients would be considered complex. The presented study extends this understanding by suggesting a structural element of complexity, from manageable to less manageable, based on ability to discharge. This spectrum suggests that teams employ strategies within the timeframe targets to manage case complexity issues and meet a discharge target – which has implications for other sectors, notably the community – but this process is not understood. Future research exploring the effect of complexity on clinician and rehabilitation practices could provide valuable insight, and may support policy and practice-level decisions, within an increasingly resource-constrained, policy-driven working environment. 

## Figures and Tables

**Table 1:** Complexity types and characteristics.

Complexity type	Complexity characteristics	
Medical/Functional	•	Severity of stroke and level of impairment
	•	Need for specialized care
	•	Comorbidities (e.g. diabetes, dialysis, visual impairments)
	•	Cognition, aphasia, dysphagia, mobility impairments
	•	Depression
	•	Pain
	•	Medication management
Social determinants	•	Education level/literacy
	•	Lack of transportation options
	•	Financial situation
	•	Access to community services
	•	Discharge setting and housing situation
Social supports	•	Community supports
	•	Family support
Personal characteristics	•	Personality/attitude
	•	Personal confidence
	•	Self-efficacy
	•	Motivation
	•	Coping skills
Health system factors	•	Disconnect between services and sectors
	•	Inclusion and exclusion criteria of other services and sectors
	•	Push to quicken discharge and decrease length of stay
